# A case-control study of physical activity patterns and risk of non-fatal myocardial infarction

**DOI:** 10.1186/1471-2458-13-122

**Published:** 2013-02-08

**Authors:** Jian Gong, Hannia Campos, Joseph Mark A Fiecas, Stephen T McGarvey, Robert Goldberg, Caroline Richardson, Ana Baylin

**Affiliations:** 1 Department of Community Health, Brown University, Providence, RI, 02912, USA; 2 Department of Nutrition, Harvard School of Public Health, Boston, MA, 02115, USA; 3 Department of Psychiatry, University of California, San Diego, La Jolla, CA, 92093, USA; 4 Department of Quantitative Health Sciences, University of Massachusetts Medical School, Worcester, MA, 01655, USA; 5 Deparment of Family Medicine, University of Michigan, Ann Arbor, MI, 48109, USA; 6 Department of Epidemiology, School of Public Health, University of Michigan, Ann Arbor, MI, 48109, USA

**Keywords:** Physical activity patterns, Myocardial infarction, Costa Rica

## Abstract

**Background:**

The interactive effects of different types of physical activity on cardiovascular disease (CVD) risk have not been fully considered in previous studies. We aimed to identify physical activity patterns that take into account combinations of physical activities and examine the association between derived physical activity patterns and risk of acute myocardial infarction (AMI).

**Methods:**

We examined the relationship between physical activity patterns, identified by principal component analysis (PCA), and AMI risk in a case-control study of myocardial infarction in Costa Rica (N=4172), 1994-2004. The component scores derived from PCA and total METS were used in natural cubic spline models to assess the association between physical activity and AMI risk.

**Results:**

Four physical activity patterns were retained from PCA that were characterized as the rest/sleep, agricultural job, light indoor activity, and manual labor job patterns. The light indoor activity and rest/sleep patterns showed an inverse linear relation (*P* for linearity=0.001) and a U-shaped association (*P* for non-linearity=0.03) with AMI risk, respectively. There was an inverse association between total activity-related energy expenditure and AMI risk but it reached a plateau at high levels of physical activity (*P* for non-linearity=0.01).

**Conclusions:**

These data suggest that a light indoor activity pattern is associated with reduced AMI risk. PCA provides a new approach to investigate the relationship between physical activity and CVD risk.

## Background

Numerous observational epidemiologic studies have demonstrated that physical activity is inversely related to cardiovascular morbidity and mortality [1-4]. Physical activity may contribute up to 20% - 30% reduced risk of coronary heart disease [5,6]. However, studies have shown that different types of physical activities may have different effects on the risk of cardiovascular disease (CVD) and may interact together [7-12]. For example, some leisure time activities such as walking, stair climbing, and cycling provide protection against CVD [7-12], whereas others, such as intensive domestic physical activity, may not offer protection against CVD [11]. There are also interactive effects between lack of exercise and sitting at work and between demanding household work and sitting at work on the association with increased risk of acute myocardial infarction (AMI) [9]. Therefore, if we use a single summary measurement to reflect physical activity, such as METS, the association between physical activity and risk of CVD might be biased because subjects who have the same measured value may have a distinct combination of physical activities. Furthermore, studying different types of physical activity in isolation may not adequately consider any joint and interactive associations on the risk of CVD.

Previous models that incorporate one type of physical activity of interest and other types of physical activity (as potential confounders) for exploring the effects of each type of physical activity on CVD may be problematic because of the concomitant change in total physical activity. As one type of physical activity increases, total physical activity increases as well, given that the other physical activities are fixed. Hence, the effect estimate of one type of physical activity does not present its pure effect, but includes the effects of total physical activity.

In order to overcome these challenges in the analysis of physical activity data, we used the method of principal component analysis (PCA) [13] to identify physical activity patterns that take into account combinations of physical activities. We used both parametric and semi-parametric regression models to examine the association between derived physical activity patterns and risk of acute myocardial infarction (AMI). Data from a population-based, case-control study in Costa Rica were utilized for purposes of this investigation.

## Methods

### Study population

In Costa Rica, CVD has been the country’s leading cause of death since 1970 and the mortality rate for CVD has been declining since 2002 according to 2007 Health in the Americas, a report from World Health Organization. The participants in this study are cases and controls from a case-control study of non-fatal myocardial infarction conducted in the Central Valley in Costa Rica from 1994 to 2004. The study design and population have been described previously [14,15]. In brief, eligible cases were men and women who were diagnosed as survivors of a first AMI by two independent cardiologists at any of the six recruiting hospitals in the Central Valley of Costa Rica during the period 1994-2004. All cases met the World Health Organization criteria for AMI [16]. Enrollment was carried out while cases were in the hospital’s step-down-unit. One free-living control subject for each case, matched for age (± 5 years), sex, and area of residence (county), was randomly selected using information available at the National Census and Statistics Bureau of Costa Rica. Participation rates were 98% for cases and 88% for controls. Cases and controls provided informed consent on documents approved by the Human Subjects Committee of the Harvard School of Public Health and the University of Costa Rica.

### Data collection

Trained interviewers visited all study participants at their homes for purposes of collecting sociodemographic characteristics, physical activity, lifestyle, medical history, smoking, and dietary data by use of a standardized questionnaire [15]. They visited cases, on average, within 3 weeks of hospital discharge (for controls, hospital discharge of the corresponding case subject) and when possible, by the same interviewer. Identical questionnaires and data collection procedures were used for cases and controls. The standardized activity questionnaire consisted of 18 questions and physical activity was determined by asking subjects the average frequency and time spent on several occupational and leisure time activities during the last year. These activities were grouped into six categories according to their intensity or metabolic equivalents (METs): lying quietly in bed: afternoon nap or rest and night sleep (0.9 METs); sitting (1.0 METs); light indoor activity such as standing at work or at home (2.4 METs); moderate outdoor activity such as gardening, light agriculture and construction, and walking on flat surfaces (3.6 METs); vigorous aerobic activity such as heavy agriculture and construction, walking uphill, climbing stairs, jogging and other sports (7.1 METs); strenuous anaerobic activity such as carrying, pushing and lifting heavy objects (7.8 METs). Energy expenditure for each activity was calculated as the product of frequency, time, and intensity (METs). Total activity-related energy expenditure per day was calculated by the sum of energy expenditure on each activity listed in our questionnaire and was measured by total METs of activity performed each day. This questionnaire was previously used in a study of 465 people conducted in Costa Rica [17,18]. The data showed that the reported time spent on different types of daily activities using the questionnaire predicted higher fitness scores, lower LDL levels, and lower BMI. These results allow us to consider that the predictive validity of the questionnaire is reasonable.

### Data analysis

All analyses were carried out with SAS (Version 9.1; SAS Institute, Cary, NC). The original sample size was composed of 2,273 cases and 2,274 controls. A total of 274 cases and 275 controls were excluded due to missing information on physical activity and the covariates in the data analysis (n=139), implausible total activity-related energy expenditure (> 2 SD from the mean energy expenditure, n=187), and losing matched controls/cases after performing rematching based on the original matching criteria (n=223). The final study sample consisted of 1999 case-control pairs (total n=3998). We used PCA on the 18 questions of the standardized activity questionnaire to identify physical activity patterns. The components (i.e. physical activity patterns) were extracted using an orthogonal matrix to achieve a simple structure that facilitates interpretability and makes the derived patterns independent of each other. The following three criteria were used to determine the number of components to retain: the criterion of eigenvalues exceeding one, the scree plot, and the interpretability of each component [13]. The component score of each pattern for each subject was calculated by summing the hours spent on physical activities weighted by their component loadings. The higher component scores indicate better adherence to a certain physical activity pattern. As part of a sensitivity analysis, we performed PCA stratified by sex.

We used paired t-tests and McNemar tests to compare means and proportions between cases and controls, given the matched design. We used parametric regression models (conditional logistic regression) and semi-parametric regression models (natural cubic splines) to assess the association of AMI risk with extracted physical activities patterns and total activity-related energy expenditure. In the parametric regression models, component scores of each extracted pattern and total activity-related energy expenditure (total METs per day) were divided into quintiles. Quintiles of those variables were entered in multivariate conditional logistic regression analysis to calculate odds ratios (OR) and 95% confidence intervals. Tests for trend were derived from conditional logistical regression with a single term representing the medians of quintiles 1-5. In semi-parametric regression models, natural cubic splines were fitted to conditional logistic regression models to examine the relationship between total activity-related energy expenditure and risk of AMI and the association between extracted physical activity patterns and risk of AMI. Natural cubic splines are smooth polynomial functions that can be used to fit data and accommodate potential changes in the direction of the association across the distribution of an exposure. They are useful to examine non-parametrically the potential non-linear relation between the exposure and the outcome of interest. They are constructed of piecewise third-order polynomials which pass through a set of control points and it is linear in its tail beyond the boundary knots [19-21]. Since they are numerically stable and allow computation of fit with great accuracy, natural cubic splines are widely used in semi-parametric regression. A SAS macro named ‘lgtphcurv9’ [22] was used which implements natural cubic spline methodology to fit potential non-linear dose-response curves in logistic regression models. Likelihood ratio tests were performed to test non-linear and linear relations [22]. In semi-parametric regression models, the median value of the first quintile of exposure was used as reference.

## Results

The baseline characteristics of the study population are shown in Table 1. Compared to controls, cases had lower annual income and higher total daily caloric intake. Cases were more likely to be current smokers, have hypertension, diabetes, hypercholesterolemia, and a sedentary lifestyle. The median total activity-related energy expenditure was 30.9 METs/day (interquartile range: 13.3) for cases and 32.4 METs/day (interquartile range: 12.9) for controls (Table 2). Cases spent more time on lying and napping compared to controls. In contrast, controls spent more time on light indoor activities and light-moderate activities (Table 2).

**Table 1:** Basic characteristics of first AMI survivors and matched controls in a case control study, Costa Rica, 1994 - 2004

**Variables**	**Cases (n=1999)**	**Controls (n=1999)**
Age (Years)	59 (11)^a^	58 (11)
Female(%)	27.5	27.5
Area of residence, % urban	39	39
Income (US$/mo)	502 (382)	570 (418)
Waist-to-hip ratio	0.97 (0.07)	0.95 (0.08)
Current smoker (%)	39.4	20.6
Hypertension (%)	39.0	30.4
Diabetes (%)	25.0	14.5
Hypercholesterolemia (%)	30.2	27.3
Dietary variables		
Total fat, % energy	32.5 (5.8)	31.9 (5.8)
Saturated fat, % energy	12.5 (3.1)	11.7 (2.9)
Monounsaturated fat, % energy	11.2 (3.5)	11.3 (4.1)
Polyunsaturated fat, % energy	6.9 (2.3)	7.1 (2.3)
Carbohydrate, % energy	54.4 (7.5)	55.4 (7.2)
Protein, % energy	13.2 (2.2)	13.0 (2.1)
Fiber (g/day)	25.1 (9.2)	24.0 (8.7)
Total calorie intake (kcal/day)	2703 (947)	2432 (751)

^a^ Mean (SD).

**Table 2:** Activity-related energy expenditure and time spent on different daily activities in a case control study, Costa Rica, 1994 - 2004

	**Activity-related energy expenditure (METs/day)**		**Time(h:min/day)**	
	**Cases**	**Controls**	**Cases**	**Control**
All physical activities	30.9 (13.3)^a^	32.4 (12.9)	21:01 (5:17)	21:30 (5:01)
Sitting	4.5 (5.5)	4.5 (5.5)	4:30 (5:30)	4:30 (5:30)
Lying and napping	1.5 (2.3)	1.2 (2.1)	1:41 (2:17)	1:17 (2:17)
Light indoor activities	10.4 (13.6)	10.7 (12.7)	4:30 (6:00)	4:38 (5:30)
Light-moderate activities	1.3 (5.2)	1.6 (5.0)	0:30 (1:37)	0:35 (1:28)
Vigorous activities	0.2 (1.3)	0.3 (1.2)	0:01 (0:10)	0:02 (0:09)
Sports	0.0 (0.0)	0.0 (1.3)	0:00 (0:00)	0:00 (0:13)
Sleeping	6.3 (1.8)	6.3 (1.8)	7:00 (2:00)	7:00 (2:00)

^a^ Median (interquartile range).

The loadings for the first four components of our PCA are presented in Table 3. The first pattern had high positive loadings on sleep measures and high negative loadings on lying in bed during the day to watch TV, read books, or listen to music, and we labeled it as the rest/sleep pattern. The second pattern had high positive loadings on items which are used to measure activities relevant to gardening and farming and high negative loadings on standing in very light activities at work or at home, and we labeled it as the agricultural job pattern. The third pattern had high positive loadings on items which are related to activities performed in the office or at home (i.e. high positive loadings on standing and moving and high negative loadings on sitting in the office or at home), and we labeled it as the light indoor activity pattern. The last pattern had high positive loadings on items which are used to assess activities related to construction (e.g. painting, chopping wood, roofing, moving or carrying heavy items, climbing steps, etc.) and high negative loadings on napping, and we labeled it as the manual labor job pattern. We performed PCA stratified by sex. There was no manual labor pattern in women, but the other three physical activity patterns were similar between women and men. Thus, we only report the results from the combined analysis to maximize power.

**Table 3:** Physical activity patterns from PCA in a case control study, Costa Rica, 1994 - 2004

	**Components**			
**Items**	**1**^**a**^	**2**^**b**^	**3**^**c**^	**4**^**d**^
Sleep during weekday	94*	2	-9	-9
Sleep during weekend	94*	-2	-7	-7
Nap	4	0	-12	-38*
Lie in bed during the day to watch TV, read, and listen to music	-45*	-7	-17	-22
Sit, either at work or in activities such as driving, watching TV	-1	-28	-74*	-12
Stand in very light activities at work or at home such as filing, coping, and doing laundry	-7	-36*	63*	-8
Stand cleaning in general such as moping, brooming, garage, washing windows, and sidewalk	4	-1	55*	-15
Standing and squatting in the garden work such as weeding and watering	2	58*	3	-3
Work in agriculture (not vigorously) such as planting, picking coffee, and cultivating.	2	58*	-6	-17
Work in construction such as painting, chopping wood, and carpentry	2	14	-13	62*
Walk on flat terrain in the city	2	17	11	15
Do heavy and vigorous jobs which made you sweat such as shovelling, digging ditches, cutting trees	3	57*	-7	9
Walk on mountainous terrain (farm)	-3	47*	5	-5
Climb steps	2	-17	-8	64*
Practice sports, i.e. teams, such as soccer, basketball, and volleyball	0	-5	-7	13
Practice sports, i.e. running, bicycling, swimming, etc.	2	-2	-5	16
Practice any other sports (not listed above)	-9	-14	-7	-13
Move or carry very heavy items which made you sweat such as carrying furniture, luggage, and water	1	12	16	31*

^a^ Rest/sleep pattern; ^b^ agricultural job pattern; ^c^ light indoor activity patter; ^d^ manual labor job pattern.

Note: Component loadings are multiplied by 100 and rounded to the nearest integer. Values greater than 0.3 are flagged by an '*'. The patterns are named based on activities that have high positive loadings, for example the agricultural job pattern have high positive loadings on activities like “standing and squatting in the garden work” or “work in agriculture”, while it has high negative loadings in activities that are more representative of other patterns (i.e. “Stand in very light activities at work or at home such as filing, coping, and doing laundry”).

Increased activity-related energy expenditure was associated with area of residence, less annual income, hypertension, higher saturated fat intake, and higher total calorie intake per day among controls (Table 4). Table 5 summarizes conditional logistic regression models that were used to evaluate the associations between four extracted physical activity patterns, total activity-related energy expenditure, and risk of AMI. The first models were controlled by matching factors (age, sex, and area of residence), and the fully adjusted models were controlled by matching factors plus adjustment for annual income, smoking status, and saturated fat intake per day. Among the four extracted physical activity patterns, only the light indoor activity pattern was significantly associated with AMI risk. As compared to subjects in the lowest level of component score, the OR for those in the highest level was 0.72 (95% CI: 0.59, 0.89; *P* for trend = 0.002) in the model adjusted for matching factors. This association remained statistically significant in the fully adjusted model (OR = 0.72, 95% CI: 0.57, 0.90; *P* for trend =0.002). However, we observed a U-shaped relationship between the rest/sleep pattern and AMI risk. In the fully adjusted model, compared to subjects in the first quintile of component score, the ORs were 0.85 (95% CI: 0.68, 1.06) for subjects in the second quintile, 0.79 (95% CI: 0.64, 0.98) in the third quintile, 0.87 (95% CI: 0.70, 1.08) in the fourth quintile, and 0.85 (95% CI: 0.69, 1.06) in the highest quintile. No statistically significant associations were found between the remaining two physical activity patterns (agricultural job and manual labor job) and risk of AMI. Total activity-related energy expenditure was negatively associated with risk of AMI. The OR for subjects in the highest vs. lowest category was 0.71 (95% CI: 0.58, 0.86; *P* for trend < 0.001) in the model adjusted for matching factors. This association did not change in the fully adjusted model.

**Table 4:** Characteristics by quintiles of total activity-related energy expenditure (METs/day) among controls in a case control study, Costa Rica, 1994 - 2004

	**Total activity-related energy expenditure (METs / day)**				
**Variables**	**Q1**	**Q2**	**Q3**	**Q4**	**Q5**
N	348	387	429	422	413
Age (Years)	60	58	58	58	57
Female (%)	24	32	31	31	19
Area of residence, %urban	52	43	39	40	25
Income (US$/mo)	601	580	595	562	515
Waist-to-hip ratio	0.96	0.94	0.94	0.95	0.95
Current smoker (%)	22	21	20	17	23
Hypertension (%)	33	32	29	34	24
Diabetes (%)	16	16	13	17	11
Hypercholesterolemia (%)	28	27	25	30	27
Plasma Triglyceride (mg/dl)	162	152	153	149	137
Plasma HDL (mg/dl)	50	50	51	52	51
Saturated fat intake (mg/day)	27	29	26	29	30
Total calorie intake (kcal/day)	2343	2442	2308	2463	2596
TEE^a^ in light indoor activities	4.0	8.8	14.2	17.0	14.0
TEE in light-moderate activities	1.5	2.1	2.5	4.2	13.2
TEE in vigorous activities	0.5	0.8	0.9	1.5	6.5
TEE in Sports	0.4	0.5	0.9	1.2	2.6
TEE in Sleeping	6.2	6.4	6.3	6.4	6.5

^a^: TEE, total activity-related energy expenditure (METs per day).

**Table 5:** Odds ratios and 95% confidence interval for AMI according to quintiles of scores for four physical activity patterns and daily total activity-related energy expenditure in a case control study, Costa Rica, 1994 - 2004

	**Quintiles of component scores for the first factor (rest/sleep)**									***P *for trend**
	**1**^a^	**2**		**3**		**4**		**5**		
Model 1	1.0	0.76^b^	0.63, 0.93^c^	0.73	0.60, 0.88	0.78	0.65, 0.95	0.82	0.68, 1.00	0.05
Model 2	1.0	0.85	0.68, 1.06	0.79	0.64, 0.98	0.87	0.70, 1.08	0.85	0.69, 1.06	0.17
	**Quintiles of component scores for the second factor (agricultural job)**									
	**1**	**2**		**3**		**4**		**5**		
Model 1	1.0	1.24	1.02, 1.50	1.36	1.11, 1.65	1.26	1.03, 1.55	1.25	1.01, 1.54	0.16
Model 2	1.0	1.11	0.90, 1.38	1.20	0.96, 1.49	1.18	0.94, 1.49	1.02	0.81, 1.29	0.83
	**Quintiles of component scores for the third factor (light indoor activity)**									
	**1**	**2**		**3**		**4**		**5**		
Model 1	1.0	0.88	0.72, 1.08	0.79	0.65, 0.97	0.79	0.65, 0.96	0.72	0.59, 0.89	0.002
Model 2	1.0	0.95	0.76, 1.19	0.87	0.70, 1.09	0.81	0.65, 1.01	0.72	0.57, 0.90	0.002
	**Quintiles of component scores for the fourth factor (manual labor job)**									
	**1**	**2**		**3**		**4**		**5**		
Model 1	1.0	0.79	0.65, 0.96	0.90	0.73, 1.09	0.79	0.65, 0.97	0.78	0.63, 0.96	0.06
Model 2	1.0	0.92	0.74, 1.14	1.01	0.81, 1.25	0.97	0.78, 1.21	0.94	0.75, 1.19	0.75
	**Quintiles of total activity-related energy expenditure (METs / day)**									
	**1**	**2**		**3**		**4**		**5**		
Model 1	1.0	0.80	0.66, 0.98	0.65	0.53, 0.80	0.67	0.55, 0.83	0.71	0.58, 0.86	<0.001
Model 2	1.0	0.80	0.64, 1.00	0.64	0.51, 0.80	0.69	0.55, 0.86	0.64	0.51, 0.80	<0.001

^a^ The first quintile as reference group; ^b^ odds ratio; ^c^ 95% confidence interval.

Model 1: adjusted for matching factors (age, sex, and area of residence).

Model 2: adjusted for matching factors, smoking status, annual income, and total saturated fat intake per day.

**Figure 1:**
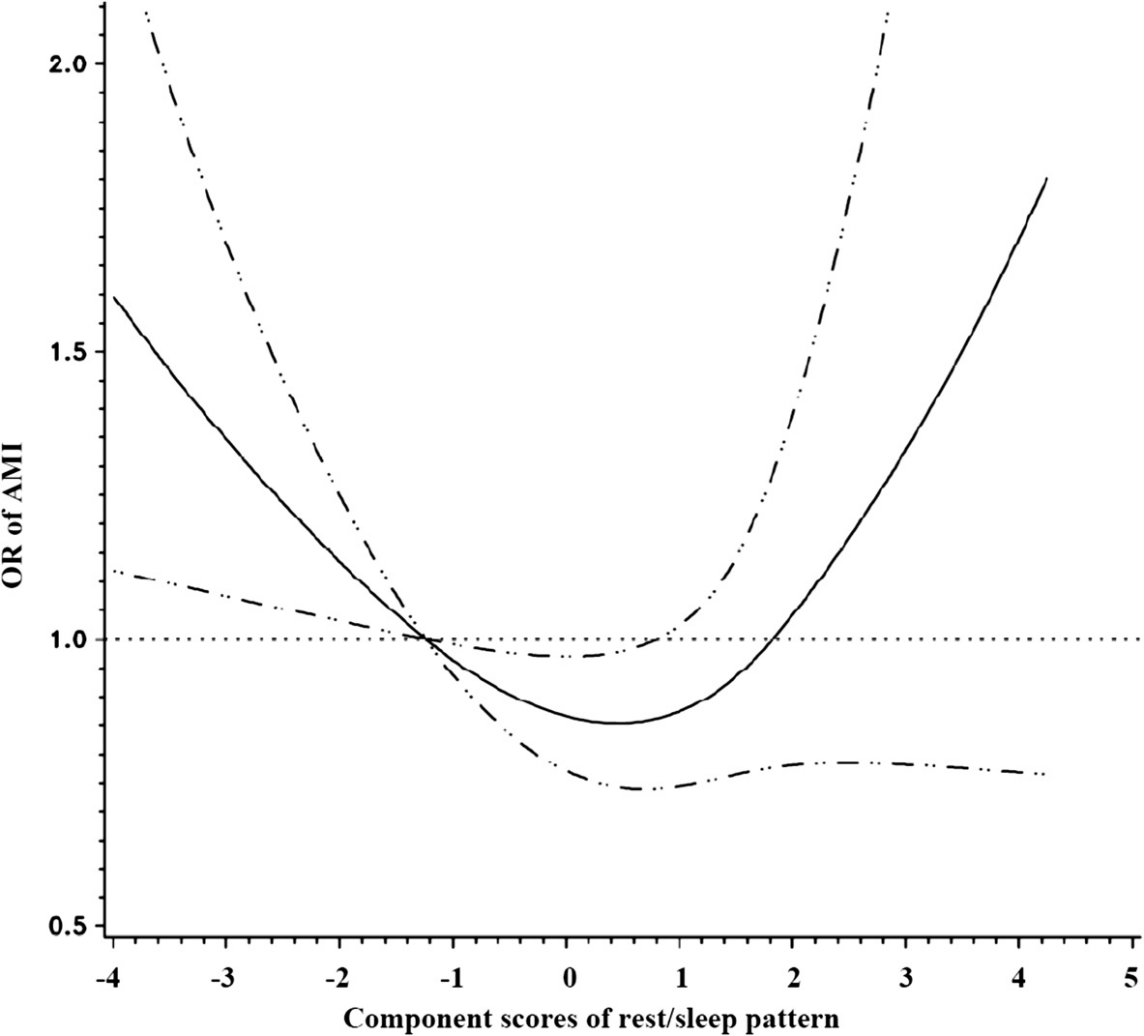
**Relationship between rest/sleep component score and risk of AMI fitted with natural cubic splines in a case control study, Costa Rica, 1994-2004.** (The reference line (OR=1.0) goes through the median value of the first quintile; the solid line for ORs; the dashed lines for 95% confidence interval boundaries).

**Figure 2:**
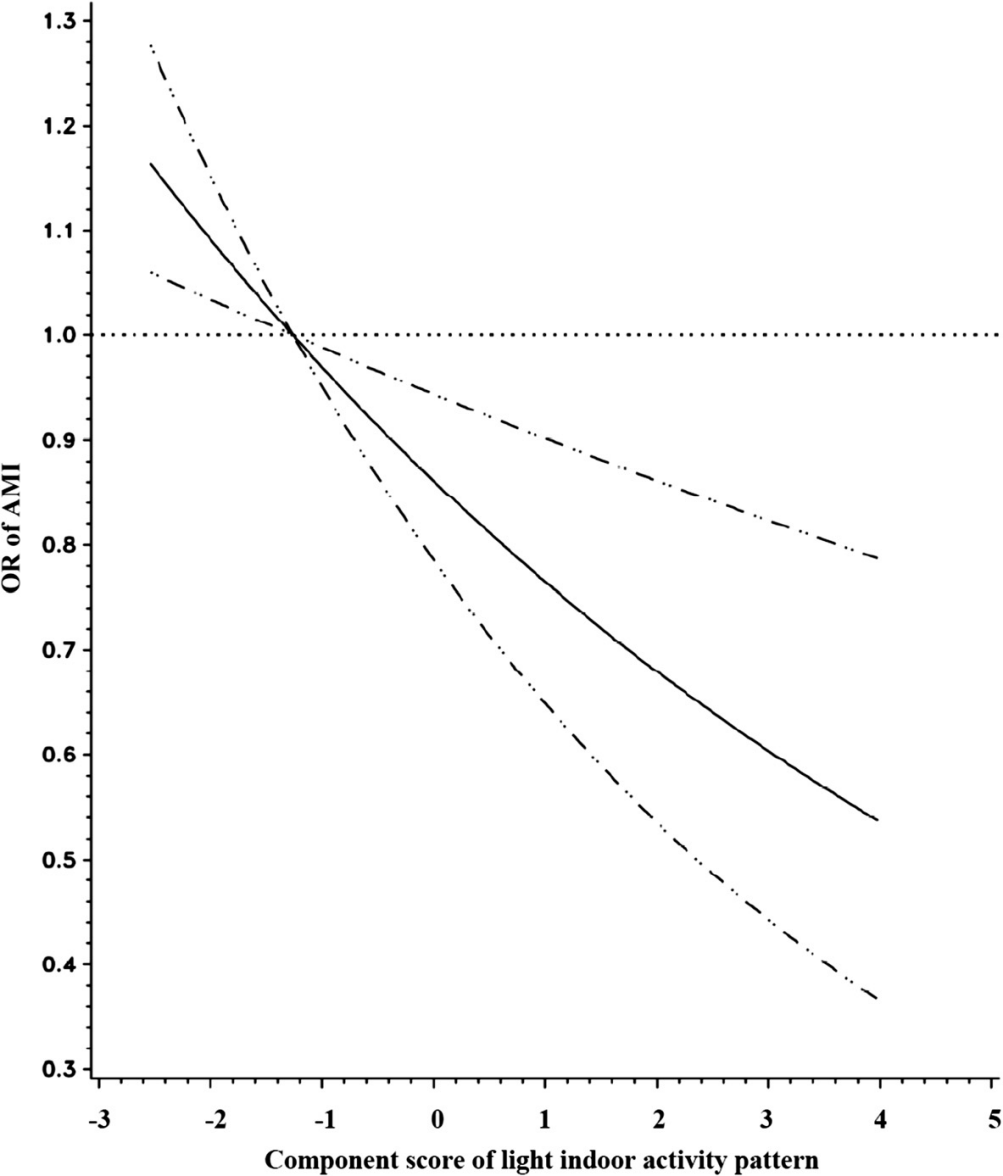
**Relationship between light indoor activity component score and risk of AMI fitted with natural cubic splines in a case control study, Costa Rica, 1994-2004.** (The reference line (OR=1.0) goes through the median value of the first quintile; the solid line for ORs; the dashed lines for 95% confidence interval boundaries).

**Figure 3:**
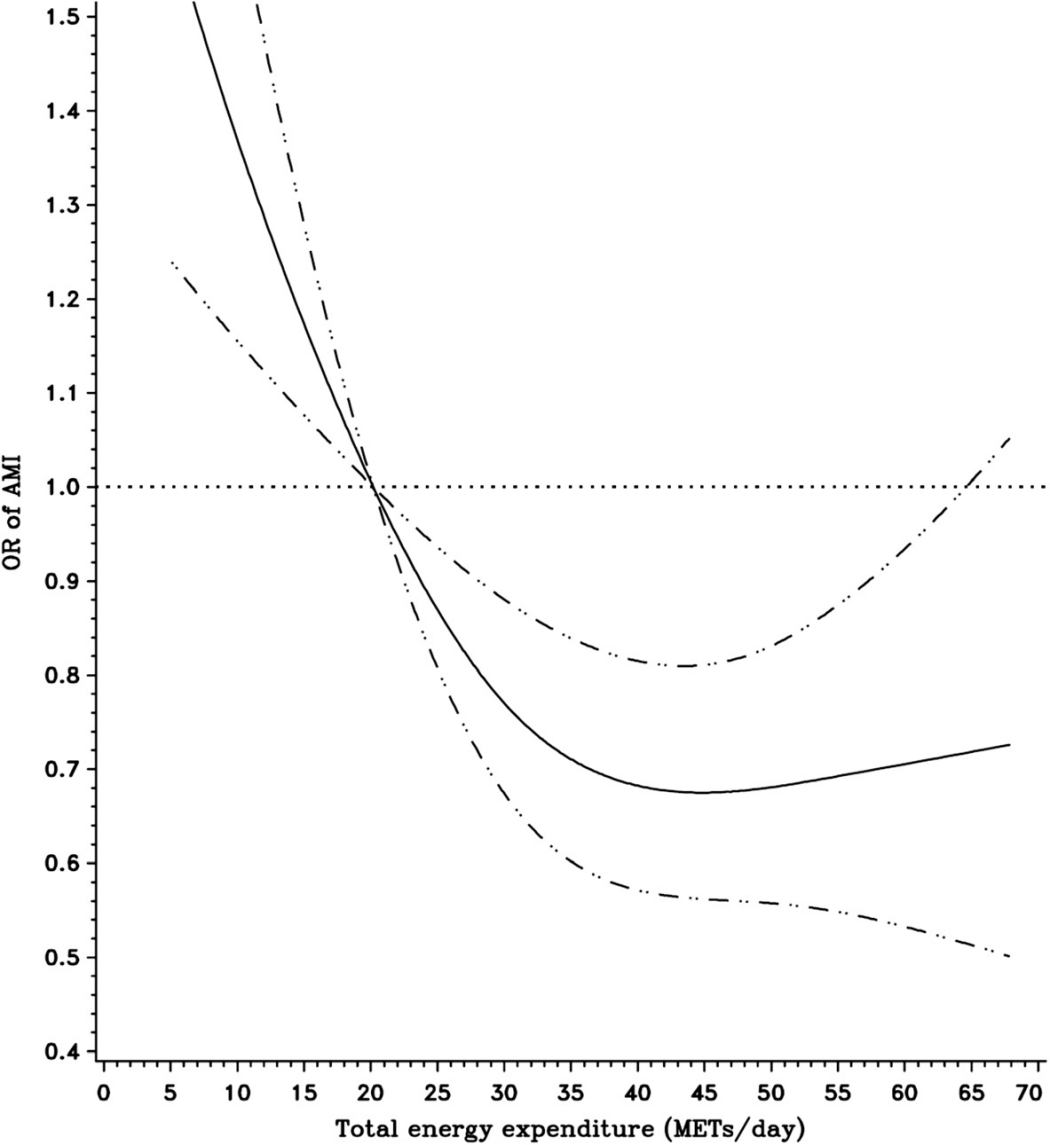
**Relationship between total activity-related energy expenditure (METs/day) and risk of AMI fitted with natural cubic splines in a case control study, Costa Rica, 1994-2004.** (The reference line (OR=1.0) goes through the median value of the first quintile; the solid line for ORs; the dashed lines for 95% confidence interval boundaries).

To further explore the association of AMI risk with the rest/sleep pattern, the light indoor activity pattern, and total activity-related energy expenditure, we fitted natural cubic splines. Models were controlled for the matching factors and potential confounders including annual income, smoking status, and daily saturated fat intake. As shown in Figure 1, there was a non linear relationship (a U-shaped relation) between the rest/sleep pattern and risk of AMI (*P* value for the non-linearity = 0.03). Consistent with the parametric models, there was an inverse linear association between the light indoor activity pattern and risk of AMI (*P* values for linear relation test = 0.001) (Figure 2). Figure 3 shows that the risk of AMI declined with the increase of total activity-related energy expenditure, but flattened out at high levels of physical activity (*P* value for the non-linearity = 0.01).

## Discussion

Four major physical activity patterns were identified from PCA in this Costa Rican population. The light indoor activity pattern was linearly and inversely associated with risk of AMI, whereas a U-shaped association was found for the rest/sleep pattern. No association was found between the agricultural job pattern and the manual labor job pattern and risk of AMI. In addition, we observed an inverse relationship between total activity-related energy expenditure and AMI risk that reached a plateau at high levels.

In this study, we utilized two approaches for exposure-response modeling: quintile presentation of the exposure and continuous presentation of the exposure fitting semi-parametric models. Compared to the former approach, the latter one has several advantages: no need for the selection of cut-points to categorize exposure, which can influence the shape of a fitted dose-response curve; no power loss; and ease of comparisons across studies [20,23]. The results from these two analytic approaches were consistent, indicating that semi-parametric models are valuable and powerful to explore the shape of an exposure-response relationship.

Previous studies have observed an association between sleep duration and risk of CVD, finding an increased risk of CHD or stroke with habitual sleeping duration of less than 6 hours per night [24-27] and long sleep duration (sleep duration >9 hours/night) [24,25]. The potential mechanisms between decreased sleep duration and risk of CHD are not fully understood but likely include sympathetic overactivity, increases in blood pressure, and decreased glucose tolerance [25]. Consistent with these results, we observed a U-shaped association between the rest/sleep pattern and AMI risk. Although the component score of the rest/sleep pattern could not provide the exact range of sleep duration beyond which the risk of AMI would be increased, the majority of the rest sleep pattern is sleeping and our results suggest that either shortened or long sleep duration could increase the risk of CHD. It is possible that longer sleep duration is related to sleep apnea [28], however we cannot assess this association directly since we did not collected sleep apnea information. On the other hand sleep duration and BMI were not associated in this population (data not shown)

Study results on the association between domestic physical activity and CVD risk vary from protective [9] to null [11]. Likewise, studies on the effects of occupational related physical activity on the risk of CVD also have shown inconsistent results ranging from protective effects [29,30] and null effects [31,32], to harmful effects [33,34]. These inconsistencies might be due to residual confounding effects, distinct definitions of domestic or occupational physical activity, measurement error, and different characteristics of the study population. In our study, the occupational physical activities in the light indoor activity pattern mainly correlated positively with standing and moving at work and inversely with sitting. These activities have been associated with a lower risk of CVD in previous studies [7,9]. On the other hand, the light indoor activity pattern did not include some strenuous or very strenuous work (e.g. lifting, carrying, and planting workload), which have been found to increase the risk of AMI [9]. We found no associations between the agricultural job pattern and the manual labor job pattern and risk of AMI. While walking and climbing steps could provide beneficial effects on CVD [9,12], some strenuous or very strenuous work such as lifting, carrying, and planting could increase the risk of AMI [9]. Thus, it is possible that the protective effects of some activities in the agricultural job and manual labor job patterns, such as walking and climbing steps, are overshadowed by the potential detrimental effects of some very strenuous activities such as lifting and carrying. It is noteworthy that agricultural and manual labor jobs in Costa Rica still include very strenuous activities as opposed to other countries like the US. On the other hand, our null findings may also be the result of measurement error and residual confounding because of imperfect adjustment for socioeconomic status and other lifestyle factors such as diet and smoking.

A dose-response relation between physical activity and risk of CVD has been well documented in several large-scale prospective studies [35-38]. However, the exact shape of the dose-response curve remains unclear. Consistent with previous studies [35-38], our study indicated that the association between total activity-related energy expenditure and AMI risk is protective. However, we observed that the decreasing risk flattened out at high levels. Occupational physical activities contributed to high levels of total activity-related energy expenditure in our study (Table 4), and we did not find an association of AMI risk with the agricultural or manual labor job patterns.

Our study has several limitations that must be kept in mind in interpreting our study findings. Our study is a case-control study and, thus, the temporal relationship between physical activity and AMI risk is unclear. As in all observational studies, we cannot establish causal associations. Self-reported physical activity measurements contain large measurement error [39,40], which may lead to underestimate the effect of physical activity on AMI risk [41]. Recall bias is an issue in case-control studies. If controls are more likely to under-report daily physical activities than cases, the results could be biased towards the null hypothesis; if controls, due to social desirability, overestimate their physical activities while cases do not, then the effects of physical activity could be overestimated. However, our results on total activity-related energy expenditure are consistent with those from previous studies. Thus, recall bias is less likely to play a role in our study. Another potential limitation is that cases only included survivors of a first AMI. We cannot exclude residual confounding in our estimates. For example, occupation stress, a potential confounder, was not accounted for in our study because the information was not available. Our results may not be generalizable to other populations, since physical activity patterns are likely to vary according to many factors such as population level economic development, individual level socioeconomic status, the built environment, and distribution of leisure and occupational activities.

## Conclusion

In conclusion, principal component analysis provides a new approach to investigate the relationship between physical activity and CVD risk and semi-parametric regression models could be a valuable method to explore exposure-response associations. Based on these approaches, we found that the light indoor activity pattern was inversely associated with risk of AMI, a U-shaped association was found for the rest/sleep pattern, and we confirmed the negative but nonlinear association between total activity-related energy expenditure and AMI risk. Further research on different populations is required to validate the application of PCA to deriving physical activity patterns and confirm our findings.

## Competing interests

The authors declare that they have no competing interests.

## Authors’ contributions

JG performed the statistical analysis and drafted the manuscript. MJAF participated in the statistical analysis. STM, RG, and CR helped to draft the manuscript. AB and HC conceived of the study, and participated in its design and coordination and helped to draft the manuscript. All authors read and approved the final manuscript.

## Pre-publication history

The pre-publication history for this paper can be accessed here:

http://www.biomedcentral.com/1471-2458/13/122/prepub
